# Klinefelter syndrome and germ cell tumors: review of the literature

**DOI:** 10.1186/s13633-020-00088-0

**Published:** 2020-09-30

**Authors:** Kimberley Bonouvrie, Jutte van der Werff ten Bosch, Machiel van den Akker

**Affiliations:** 1 Department of Pediatrics, Maxima Medisch Centrum, Veldhoven, The Netherlands; 2 Department of Pediatrics, ZNA Queen Paola Children’s Hospital, Lindendreef 1, 2020 Antwerp, Belgium; 3 Department of Pediatric Hematology and Oncology, University Hospital Brussel, Brussels, Belgium

**Keywords:** Klinefelter syndrome, Extragonadal germ cell tumor malignancy, Incidence analysis

## Abstract

**Objective:**

The most common presentation of Klinefelter syndrome (KS) is infertility and features of hypogonadism. Currently no consensus exists on the risk of malignancy in this syndrome. Several case reports show an incidence of extragonadal germ cells tumors (eGCT) of 1.5 per 1000 KS patients (OR 50 against healthy population). Malignant germ cell tumors are rare in children. They account for 3% of all children cancers. Young patients with a germ cell tumor are not routinely tested for Klinefelter syndrome. This can therefore result in underdiagnosing. Literature data suggest a correlation between eGCT and KS. To the best of our knowledge there is no precise description of the primary locations of germ cell tumors in KS patients. The purpose of this study is to evaluate age groups and primary locations of extragonadal germ cell tumors in Klinefelter patients. With this data we investigate whether it is necessary to perform a cytogenetic analysis for KS in every eGCT patient.

**Study design:**

This study is based on case report publications in PubMed/Medline published until march 2020 that described “Klinefelter Syndrome (MeSH) AND/OR extragonadal germ cell tumors”. Publications were included when patients age, location and histology of the germ cell tumor was known. Two double blinded reviewers selected the studies.Results: 141 KS patients with eGCTs were identified. Mean age at presentation was 17.3 years (StDev + − 10.2). In contrast to the extragonadal germ cell tumors in adults, most eGCT in children were mediastinal or in the central nervous system (respectively 90/141; 64% and 23/141; 16% of all tumors). Distribution of histologic subtypes showed that the largest fraction represented a teratoma, mixed-type-non-seminomateus GCT and germinoma, respectively 34/141; 24%, 26/141; 18% and 20/141; 14% of all tumors.

**Conclusion:**

These data suggest a correlation between primary extragonadal germ cell tumors and Klinefelter syndrome. There appears to be an indication for screening on KS in young patients with an eGCT in the mediastinum. A low threshold for radiologic examinations should be considered to discover eGCT. We emphasize the need for genetic analysis in all cases of a male with a mediastinal germ cell tumor for the underdiagnosed Klinefelter syndrome.

## Introduction

Klinefelter syndrome (KS) is characterized by hypogonadism, gynecomastia, infertility and the addition of at least one extra X chromosome to the standard human male karyotype (most frequently 47,XXY) [1]. KS is usually acquired through a non-disjunction during parental gametogenesis of either paternal (53%) or maternal (44%) origin [2]. The greater the number of extra X chromosomes (47 XXY or a mosaicism), the greater the phenotypic consequences, both gonadal and extragonadal [3]. KS is the most common sex chromosome disorder, occurring in about one out of 600 males [4]. Tough only approximately one fourth of adult males with KS are diagnosed [5]. KS has been associated with conditions like venous disease, auto-immune disorders, mild neurobehavioral deficit, diabetes mellitus, sexual precocity and osteoporosis [6]. KS patients have an increased risk of several malignancies, especially male breast cancer [7, 8] and extragonadal germ cell tumors, primarily localized in the mediastinum. Male breast cancer has been most highly associated with the 47 XXY mosaics, suggesting up to 20–30 times greater incidence in patients with KS compared to patients with normal karyotype [7, 8].

Different neoplasms such as testis, lymphoreticular malignancies may occur in 1–2% of the cases with KS. Hematological malignancies, such as leukemia and lymphoma have been described as well [9].

KS has been described earlier in correlation with an increased risk of developing extragonadal germ cell tumors, though routine screening in the KS population is not standard of care [10]. The most common primary site for an extragonadal germ cell tumor (eGCT) is the mediastinum, followed by the peritoneum [11]. GCTs are tumors originating from the germ cells, the precursors of the sperm and ova, and have the potential to produce all of the somatic (embryonic) and supporting (extraembryonic) structures of a developing embryo. They account only for 3.4% of all pediatric malignancies [12]. Germ cell tumors are the most common malignancy of the testicle, with a peak incidence in males between 15 and 35 years old. It is known that about 5% of the germ cell tumors are extragonadal [13]. A large number of eGCT have been described in association with KS, most often located in the mediastinum with a relative risk of at least 50 [14]. There are different risk factors available in literature. Williams et al. describe that approximately one-third of males with mediastinal germ cell tumors have Klinefelter syndrome [15], while others describe only 8% of male patients with primary mediastinal GCT have KS [14]. The overall incidence of cancer in men with KS seems to be similar to that of the general population but some malignancies show a significantly higher prevalence. Although data in the literature on cancer prevalence in KS are abundant, most of them are individual case reports. By reviewing all cases of KS and GCT reported in the medical case reports, we show the relationship between KS and eGCT, and thereby emphasizing the need for genetic analysis in all cases of a male with a mediastinal GCT for the underdiagnosed KS.

## Methods

PubMed/Medline search was conducted in the English-language literature using the keywords “Klinefelter Syndrome (MeSH) AND/OR extragonadal germ cell tumors”. The literature was reviewed from 1967 until march 2020 on eGCT associated with Klinefelter syndrome. All patients with KS and an eGCT were documented by author of the paper, year of publication, location of primary tumor, age of the patient and histology of the tumor. The locations of the germ cells tumors were divided into; mediastinum, central nervous system, testis and abdomen. The major morphologic categories are embryonal carcinoma, germinoma, teratoma, endodermal sinus tumor (yolk sac), choriocarcinoma and gonadoblastoma. Teratocarcinoma refers to a germ cell tumor that is a mixture of teratoma with embryonal carcinoma or choriocarcinoma, or with both. When not further specified, cases are classified under mixed GCT of unknown origin.

## Results

A total of 196 papers were reviewed and 147 of patients with KS and a GCT identified. Two patients presented with a double tumor and were excluded in further analysis: a patient with both testis and mediastinum tumor and an 8-year-old patient with a hypophyseal stalk and mediastinum tumor. Of 145 patients 4 persons had unknown ages and were excluded for further analysis. A total 141 patients were included in this analysis.

### Articles published per year

Articles that were included were published from 1972 until march 2020. From 1971 until 1986 only several reports were written (total of 38 patients). Most patients found in case reports (total 72 patients; 51%) were published between 1987 and 2006. Since 2006 30 patients with KS and GCS were published in several case series.

### Age of the patients

**Fig. 1:**
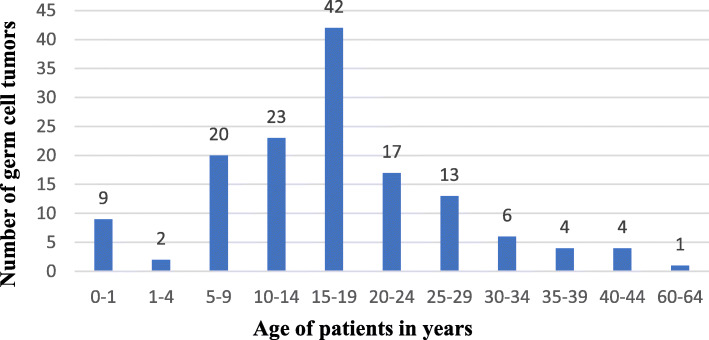
Germ cell tumor incidence in Klinefelter syndrome by age group (*n* = 141)

Mean age was 17.3 years (StDev + − 10.2). Thirty percent (42 cases) of the reported cases were in the age-group of 15–19 years old. Of 141 patients, 54 patients were below the age of 14 (prepubertal), as 87 patients were postpubertal with the oldest age of 64 years. The incidence of germ cell tumors by age group are represented in Fig. 1.

### Localization of the tumor

**Fig. 2:**
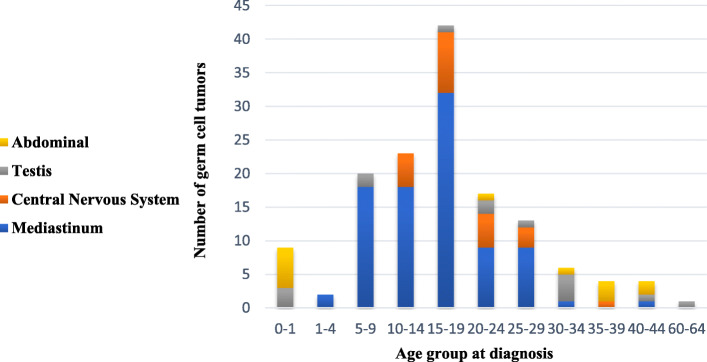
Germ cell tumor indicence by location and age group. Most germ cell tumors in klinefelter patients were diagnosed were reported between the age of 15–19 years. The mediastinum is the most common location in age group 5–29 years

The most frequent location of the germ cell tumors appeared to be the mediastinum (*n* = 90), followed by the central nervous system (*n* = 23), testis (*n* = 15) and abdomen (*n* = 13) (Table 1). Especially in age 5–19 years the mediastinal germ cell tumors comprise the largest fraction (Fig. 2). Children under the age of 5 show more intra-abdominal localizations (*n* = 6). The central nervous system seems to be more involved at postpubertal age (15–29 years old).

**Table 1:** Localization of extragonadal germ cell tumors of 141 Klinefelter syndrome patients. Most germ cell tumors were found in the mediastinum, followed by the central nervous system

	Total patients (n)	% patients
**Mediastinum**	90	64
**Cental Nervous system**	23	16
**Testis**	15	11
**Abdomen**	13	9
Total	141

#### Histology of the tumor

**Fig. 3:**
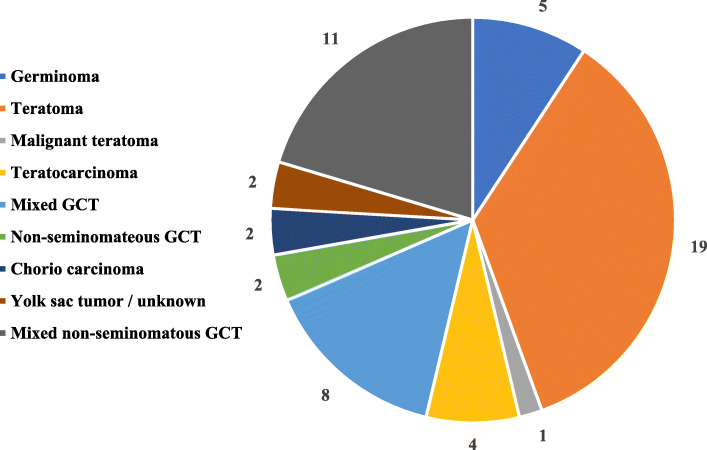
Germ cell tumors in prepubertal patients (*n* = 54, age 0–14 years). Most common germ cell tumors below the age of 14 years found is a teratoma, followed by an mixed non-seminomatous germ cell tumor. * GCT = germ cell tumor

**Fig. 4:**
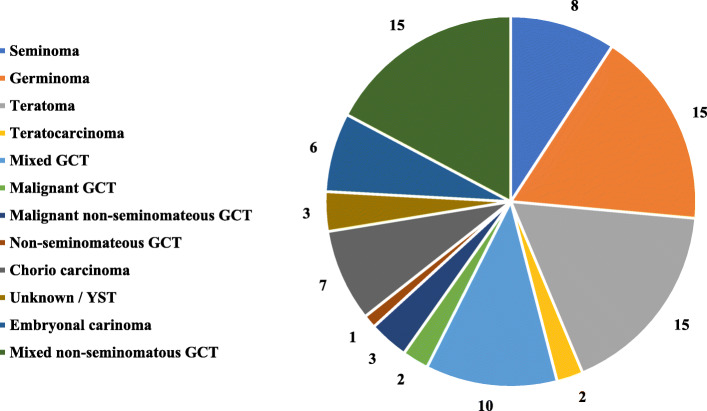
Germ cell tumors in postpubertal patients (*n* = 87, age 15–64 years). Most common germ cell tumors above the age of 15 years found is a teratoma, germinoma and an mixed non-seminomatous germ cell tumor. * GCT = germ cell tumor. * YST = yolc sac tumor

Distribution of histologic subtypes showed that the largest fraction represented a teratoma, mixed-type-non-seminomateus GCT and germinoma, respectively 34/141; 24%, 26/141; 18% and 20/141; 14% of all tumors. Figure 3 show the prepubertal Klinefelter patients were the largest fractions of mediastinal germ cell tumors comprise teratomas (*n* = 19), mostly found in age 0–1 years (*n* = 9) and 5–9 (*n* = 7). Secondly 11 non-seminomatous GCT were found in prepubertal patients, most in age 10–14 (*n* = 8). Thirdly, eight mixed germ cell tumors were found, mostly in age 5–9 years (*n* = 4) and 10–14 years (*n* = 3). Between the ages of 5–9 years old there is a mix of teratomas, mixed GCTs and mixed non seminomatous GCT. In age group 10–14 the mixed non-seminomateus GCT’s with germinomas and mixed GCTs are the most frequent. In the largest group of 15–19 years of age there is a mix of mixed non-seminomatous GCT, teratomas, malignant GCT, germinomas and yolk sac tumors. From the 87 postpubertal patients the largest fractions of mediastinal germ cell tumors comprise germinoma (*n* = 15), teratoma (n = 15) and mixed non-seminomatous GCT (*n* = 15) all most found in age 15–19 years (Fig. 4).

## Discussion

### Etiology

Tumors of germ cell origin occur with increased frequency in disorders of sexual differentiation, including cryptorchidism, XY dysgonadal dysgenesis, testicular feminization syndrome and KS. The overall incidence of cancer in men with KS is similar to that of the general population, but some malignancies show a significantly higher prevalence in these patients. The reported incidence of eGCT in KS patients is 1.5/1.000, a fifty-fold increase over the general population [16]. Although it has been estimated that 1–2% of KS patients develop a neoplasm, previous data demonstrated that young males with KS have a relative risk of around 66.7 for developing extra-gonadal germ cell tumors [17, 18]. Several reports describe the association between KS and GCT, although the cause of this association is unknown. Some postulate that the hypergonadotropism due to the gonadal insufficiency promote germ cell proliferation, while others hypothesize the assignment of a GCT susceptibility gene to the X chromosome [19]. In KS is the mediastinum the most prevalent site compared to the gonads in those with KS. Until now the altered hormones, such as elevated estrogen-to-testosterone ratio and elevated gonadotropin level have been the focus for explaining the observed increase in male breast cancer and extragonadal germ cell tumors among Klinefelter syndrome patients [7, 20]. The abnormal hormonal influence could also increase the malignant potential [10].

### Localization and age

GCTs originate from primitive germ cells, which migrate during the embryogenesis from their origin in the endoderm in the yolk sac along the urogenital ridge to the gonads. Abnormal migration causes the germ cells to be distributed, to the gonads and to extragonadal localizations. In the normal course of development these cells die off, but if they are persistent or misplaced, they may give rise to germ cell tumors. This may be the result of an abnormality in the primordial germ cell itself or in its micro-environment [21]. Furthermore, there is a hypothesis that the germ cells transformed in the testis have a reserve migration pathway [22]. This hypothesis is supported by the fact that testicular GCTs and ECTGs share the common cell of origin [23, 24]. Germ cell tumors are classified as extragonadal if there is no evidence of a primary tumor in the testis [25]. By unknown factors, dislocated germ cells undergo malignant transformation and to give rise to GCTs in extragonadal localizations. Germ cell tumors arise in both gonadal and extragonadal sites. In adults, 90% of primary germ cell tumors involve the testis or ovary, but it is known that in the pre-pubertal years there is a preference for extragonadal sites [26]. It is known that most primary eGCTs are found in midline structures (pineal region (6%), mediastinum (7%), retroperitoneum (4%), sacrococcygeal region (42%) or in the ovary (24%), testis (9%), and other sites (8%) [27]. The anterior mediastinum is the third most common location for germ cell tumors in children. The mediastinum gives a generous space for expansion before the mass will cause symptoms, mediastinal germ cell tumors may achieve enormous size prior to detection. The patient’s age at diagnosis appears to be a critical prognostic factor. EUROCARE data (European Cancer Registry based study on survival and care of cancer patient’s project) stated that GCTs have an incidence of 31/1.000.000 people. Most GCTs arise in the gonads, with the highest incidence between age 25–40 years (testis) and 14–34 years (ovarian). eGCTs occur most frequently in the age-groups 0–4 years and 15–34 years. It is assumed that the most common extragonal localizations in children are the central nervous system, followed by mediastinum/thorax and abdomen/pelvis. In our study more eGCTs were found in the mediastinum than in the central nervous system. The most common localization of a GCT in KS, is the mediastinum (*n* = 83) except for children under the age of 5 year in which the abdominal location is more frequent (*n* = 8). This is comparable with other known data. The GCTs are mostly located in the anterior mediastinum [28, 29].

In adults without KS, GCTs are mostly gonadal, whereas in children without KS, GCTs are mostly extragonadal (CNS responsible for the primarily location). Beresford et al. proposed that chest Rx should be performed as routine in young patients with KS [30]. The risk to develop a mediastinal GCT for children with KS is difficult to assess due to the rarity of mediastinal GCT and the underdiagnosed KS.

### Pathology

Extragonadal germ cell tumors (eGCTs) represent 5–10% of all GCTs and most arise in the midline [31]. The most common midline sites are mediastinal, retroperitoneal, pineal gland and sacrococcygeal [32]. They can be divided into two histologic groups, germinoma and non-germinoma. For a tumor to be considered in one group, it must entirely consist of that single histology and cannot contain other GCT elements. The germinoma group is divided in germinoma (most often brain), dysgerminoma (ovary) and seminoma (testis), while the non-germinoma group is divided in teratoma (mature and immature), yolk sac tumor (endodermal sinus tumor), choriocarcinoma, embryonal carcinoma, gonadoblastoma and mixed GCTs. Seminomas may exist in a pure form, but any elevation of AFP indicates the presence of an element of a non-seminomatous tumor [33]. In addition, mediastinal germ cell tumors have a propensity to develop another malignancy (e.g., rhabdomyosarcoma, adenocarcinoma, permeative neuroectodermal tumor), which can become the predominant histology. Determination of serum tumor markers, β-HCG and AFP, is important in the diagnosis and follow-up of germ cell tumors. AFP and or beta-hCG is elevated in 85% of cases of extragonadal non-seminomatous GCTs [34] [35]. Elevations of β-HCG and AFP confirm a malignant component to the tumor. Yolk sac tumors produce AFP, while germinomas and choriocarcinomas, produce β-HCG. Benign teratomas may produce small elevation of the tumor markers.

## Conclusion

Our data suggest there is a strong correlation between primary germ cell tumors and Klinefelter syndrome. A higher prevalence of germ cell tumors in KS patients is seen in the mediastinum of young adults. We recommend that there is an indication to perform genetic analysis to confirm Klinefelter syndrome in young patients with an extragonadal germ cell tumor in the mediastinum.

Screening every patient with Klinefelter syndrome for early detection of germ cell tumors can be deliberated. Patients with Klinefelter syndrome should be closely monitored and provided with adequate information on the risk of malignancy.

## Supplementary information

**Additional file 1.**

## Data Availability

All data generated or analysed during this study are included in this published article and its supplementary information file.

## Abbreviations

KS: Klinefelter Syndrome
eGCT: Extragonadal germ cell tumors
GCT: Germ cell tumors
CNS: Central nervous system
beta-hCG: Beta human chorionic gonadotropin
StDev: Standard deviation
